# How to Implement Game-Based Learning in a Smart Classroom? A Model Based on a Systematic Literature Review and Delphi Method

**DOI:** 10.3389/fpsyg.2021.749837

**Published:** 2021-12-02

**Authors:** Liuxia Pan, Ahmed Tlili, Jiaping Li, Feng Jiang, Gaojun Shi, Huiju Yu, Junfeng Yang

**Affiliations:** ^1^Jing Hengyi School of Education, Hangzhou Normal University, Hangzhou, China; ^2^Smart Learning Institute, Beijing Normal University, Beijing, China; ^3^School of Marxism, Hangzhou Normal University, Hangzhou, China

**Keywords:** games-based learning, smart classroom, teaching model, smart learning environment, education game

## Abstract

Game-based learning (GBL) can allow learners to acquire and construct knowledge in a fun and focused learning atmosphere. A systematic literature review of 42 papers from 2010 to 2020 in this study showed that the current difficulties in implementing GBL in classrooms could be classified into the following categories: infrastructure, resources, theoretical guidance, teacher’s capabilities and acceptance of GBL. In order to solve the above problems, the study constructs a technology enhanced GBL model, from the four parts of learning objective, learning process, learning evaluation, and smart classroom. In addition, this study adopted the Delphi method, inviting a total of 29 scholars, experts, teachers and school managers to explore how to implement GBL in smart classrooms. Finally, the technology enhanced GBL model was validated and the utilization approaches were provided at the conclusion part.

## Introduction

Game-Based Learning (GBL) originated from the game research in the middle of the 1950s, and from the 1980s scholars started the research and practice of integrating games into instruction. With the popularization of electronic games and the transformation of education concepts, people gradually started accepting games as learning tools (Seaborn and Fels, 2015). The published papers on WoS (Web of Science) tagged by “Game-Based Learning” have demonstrated a rapid increase and interest in this field.

GBL refers to applying games or related elements, concepts, mechanisms or designs into learning (Deterding et al., 2011), which is a study mode that integrates educational games into school teaching and self-regulated learning. As a result, learners can get immersive learning experiences while mastering knowledge and skills.

GBL has been applied into classroom teaching. However, in terms of practice, there are still some problems, such as lack of integration between gaming and teaching, a poor balance between the enjoyment effect, and the education effect. Games are either too attractive but failing to reflect studying goals, or games can be too educational but failing to trigger interests among learners (Zhang and Liu, 2007). Some educational games simply provide learning content in a digitalized way, emphasizing memorizing facts (Villalta et al., 2011). Apart from that, being constrained to the equipped devices and internet condition of the classroom, the effect and experience of games is much less satisfying (Shin and Chung, 2017; Halloran and Minaeva, 2019). Sometimes, due to the hardware conditions, applications of digital games have to be forgone. Many scholars and enterprises conducted related design and research of digital educational games, but its practical application is hard to meet the requirements of related studying activities because of the location, equipment, and internet (Xuqing, 2007; Hou et al., 2012). It is clear that learning resources, classroom environment and technical configuration play a vital role in the implementation of GBL (Dickey, 2011; Sabourin and Lester, 2013). However, lots of problems exist to carry out GBL in classrooms.

With the advance of educational technology, the research and practice of smart classroom became popular since 2012 (Yang et al., 2018), which utilized digital technology to support flexible pedagogies, including GBL. The smart classroom is a type of technology-enhanced classroom space to facilitate content presentation, class management, learning resources accessing, and instructional interaction by utilizing appropriate devices and software (Huang et al., 2012). With the development of research and practice on smart education, it is possible to carry out GBL in smart classrooms to overcome the above-mentioned problems.

In a smart classroom, with GBL, students could engage in learning by using quality game resources via digital or VR devices with broadband Internet access, hence enhance the digital GBL experience. Therefore, this study aims to promote GBL in classrooms by utilizing smart classroom. Specifically, this study answers the following two research questions:

(1) What are the problems of implementing GBL in classroom?
(2) How to implement GBL in smart classrooms?

## Related Work

### Related Concepts of Game-Based Learning

Games can be divided into many different categories based on form and content (Amory et al., 1999; Tian et al., 2018). For conducting GBL research, the following three terms are always mentioned, namely “Serious game,” “Educational game,” and “Digital educational game.” There is a certain connection intersection and difference between these three terms. Clarifying the meanings and relationships of these three types of games can determine the scope of the game in this study more clearly. In this study, GBL is considered as a type of educational activity based on digital educational games, which can also be understood as digital game-based learning (DGBL) (Perini et al., 2018; Chen et al., 2020).

The term “serious game” was first used by Abt to describe games designed for learning (Apt, 1970). In particular, Abt stated that serious games must have an educational purpose and not be played primarily for entertainment. Serious games (Apt, 1970) can teach players knowledge and skills, and at the same time, provide professional training and simulation. Serious games have a proven ability to facilitate the development of skills, abilities and attitudes due to their focus on problem-solving, to which players are exposed (The Gamification of Learning and Instruction, n.d.; Ritterfeld et al., 2009). The content of serious games involves personnel training, policy discussion, military, education, health, medical treatment, etc.

Educational games are games explicitly designed for education (Amory and Seagram, 2003; Ahmad et al., 2015). It includes both physical and digital games. Educational games in a narrow sense are electronic games specially developed for educational purposes (Moreno-Ger et al., 2008; Habgood and Ainsworth, 2011). Educational games in a broad sense not only involve traditional games (Vos et al., 2011) (such as origami, seven-piece puzzle, messaging game, etc.), but also include all educational software, teaching aids, toys with both the characteristics of education and fun, for example, electronic game tables developed for educational use, commercial games with educational value, and some interesting educational software, etc. Educational games should be developed by considering the objectives and functions of education.

Digital educational games (also referred sometimes as educational video games) are educational games which are digital (Law and Sun, 2012). From the perspective of participating in games, digital educational games need information technology equipment and various digital platforms to support the development of games (Lin and Lin, 2014; Aslan and Balci, 2015; Hawlitschek and Joeckel, 2017). Digital educational games also need to meet educational features, which can promote learners’ understanding of the learning content. There are several types of digital educational games, including adventure and role-playing games, business games, board games, combat games, logic games and puzzles, and word games (Alessi and Trollip, 2001), and digital educational games may be designed for single player (Miller et al., 2011) and multi-players (Annetta et al., 2009).

### Advantages of Game-Based Learning

GBL is often characterized as more fun, engaging, moving, and symbiotic (Brangier and Marache-Francisco, 2020; Osipovskaya and Miakotnikova, 2020; Tundjungsari, 2020). GBL allows learners to participate in authentic learning environments, providing a fun, interactive and challenging learning environment while enabling learners to experience and apply knowledge (Chen et al., 2018). GBL provides learners with a contextualized and personalized learning environment (Sykes and Dubreil, 2019) that meets the individual needs of different types of learners.

GBL is a type of educational game that improves students’ attitudes and approaches to learning and allows them to appreciate the learning process itself (Yadav and Oyelere, 2020). Many studies have shown that digital game-based learning has a positive impact on learners’ motivation, attitude (Tapingkae et al., 2020; Taub et al., 2020), engagement and performance (Eltahir et al., 2021). The use of game elements, such as levels, points, leaderboards and competitive environment, can not only promote students’ external motivation, but also positively affect students’ behavior and increase their internal motivation in subjects and concepts that are difficult for students to understand (Kalogiannakis et al., 2021). GBL uses game elements and aesthetics to enhance students’ motivation and promote learning (Zimmerling et al., 2019). Appropriate competition and challenge can motivate learners to learn. Games often have game mechanics such as competition, scoring, and ranking that motivate learners to win, gain a sense of accomplishment and satisfaction, and make learners highly motivated to learn (Jagušt et al., 2018).

GBL not only has a positive impact on student’s learning, but also increases their self-efficacy (Wang and Zheng, 2020). Digital games with interesting storylines, clear objectives and tasks to be solved make teaching and learning more diverse and effective in increasing students’ interest and learning efficiency (Yang and Lu, 2021).

GBL not only engages learners in learning, but also deepens their understanding of textbook content so they can solve more complex problems (Perini et al., 2018). Learners can explore games and find different solutions to problems; therefore, creative thinking and critical thinking can be developed (Nadolny et al., 2020). Learners can explore the game and find different problem solutions; thus, the creative thinking and critical thinking could be trained (Amory et al., 1999; Nadolny et al., 2020).

### Theoretical Foundations for Game-Based Learning

In this research, 16 relatively high-quality research reviews in the last 5 years have been searched from the major databases in this field (Web of Science, EBSCO ERIC (Education Resources Information Center), IEEE Xplore and SpringerLink). After reviewing these papers, it is found that their main concerns can be summarized into the following four aspects: the effectiveness of GBL (Meredith, 2016; Byun and Joung, 2018; Hussein et al., 2019; Pellas et al., 2019; Tokac et al., 2019; Chen et al., 2020; Garcia et al., 2020; Karakoç et al., 2020; Stančin et al., 2020), the future trend of GBL (Giannakas et al., 2018; Gao et al., 2020), the influencing factors of GBL (Perttula et al., 2017; Shu and Liu, 2019), the theoretical foundations of GBL’s effectiveness and its practical use (Carenys and Moya, 2016; Bakan and Bakan, 2018; Ab Jalil et al., 2020).

After synthesizing some literature reviews of predecessors, this research found that there is relevant theoretical support for GBL. Some studies suggest that the theories underlying GBL studies can be classified into three categories: learning theories, motivational theories, and others (Carenys and Moya, 2016). The behaviorism, cognitivism, humanism and constructivism (Amstutz, 1999; Guy, 1999; Merriam, 2001; Conole et al., 2004). Learning theories are the basis for the development of propositions in GBL. Each learning theory has its own representative principles, which provide theoretical guidance for GBL.

According to behaviorism, players need to know their goals and achieve these goals through stimuli–reaction process (Wu et al., 2012). Cognitivists consider learning not to be simply stimulation and reinforcement, but to involve thinking (Moore and Fitz, 1993). Cognitivism emphasizes the context-dependent nature of knowledge where learning is promoted through scaffolding for task completion. Humanism emphasizes that the learner-centered approach is the most important component and players can play games at their own pace and according to their mood (Kolb, 2014). Constructivism is probably the learning theory that offers propositions closest to GBL (Carenys and Moya, 2016). It states that learners must be provided with the tools that allow them to construct their own body of knowledge and that instructors should be facilitators who accompany them in this self-learning process. These statements are strongly linked to the learner-centered education model and the active learning proposed by GBL. In the part of model construction, this study refers to the input-process output model (Garris et al., 2002), the Play Curricular activity Reflection and Discussion (PCaRD) GBL pedagogical model (Denham, 2019), and the ARCS model (Keller, 1987).

### Affordance of Smart Classroom

There has been a large amount of work on smart classrooms spanning over a wide range of research areas including information communication technology, machine learning, sensor networks, mobile computing, hardware (Lämsä et al., 2018). From the educational perspective, smart classrooms should integrate physical and virtual environments to provide blended environments for learners.

The physical environment of smart classrooms includes convenient learning facilities, high-speed Internet access, comfortable surroundings, flexible space layout, etc. (Paternò and Wulf, 2017). Convenient learning facilities include various types of learning terminals, display terminals, and real recording terminals, which can effectively support the presentation and sharing of learning content and learning results, and support the communication and interaction between teachers and learners. Smart classrooms have high-speed Internet access, equipped with relatively complete network communication facilities, including wired communication devices, wireless communication devices, stable and efficient server and controller. This can ensure a fluent game process and communication, allowing learners to have a good gaming experience. This can also allow multiple devices to operate stably at the same time to meet the requirements of all learners’ participation. In order to provide learners with a comfortable classroom, sensing systems are installed in the classroom, which can control the temperature, light, sound and air quality (Torrente et al., 2008). The flexible spatial layout is mainly to provide learners with a more open venue for activities, rather than confining the space for teaching activities to closed conventional rooms (Brezovszky et al., 2019). Desks and chairs with humanized designs are provided so that learners can change their positions according to their needs, and form learning groups to facilitate teamwork and group learning activities. In addition, it also includes other related equipments that can meet the needs of teaching and learning activities, such as printing equipment, multimedia editing equipment, bookcases, shelves, etc.

The virtual environment of smart classrooms, based on cloud platforms, cloud servers, cloud computing, cloud storage, etc., is normally equipped with corresponding cloud diagnostic analysis systems to build a virtual learning space. From the perspective of learning, the virtual environment of smart classrooms should provide the functions of learning context-aware, connecting learner’s community, accessing learning resources, and personalizing learning pace (Denham, 2019). When environmental or user parameters are changing, classrooms with context awareness are able to determine the reactions based on certain rules or AI algorithms (Fang and Strobel, 2011; Allsop and Jessel, 2015). Social networking, e-learning spaces, internet and other technologies in a smart classroom connect learning participants and bridge the communication between teachers and learners, allowing to extend the interaction beyond classrooms (Chen et al., 2020), which promotes the construction of a learning community. Another important element of a smart classroom is the abundance of learning resources. The digital resource platform integrates a large amount of online data and materials for learners, and manages them by category to help learners obtain high-quality learning resources more conveniently (Denham et al., 2016). In addition, learners’ personalized learning is also an essential element (Belova and Zowada, 2020). The management system in the smart classroom can provide services and feedback to learners, so that they can adjust and manage the learning pace as needed, which can promote their self-regulated learning.

According to the above sorting out of the characteristics of virtual environment and physical environment in smart classrooms, the functions of smart classrooms are as follows: (1) The learning content is flexible and diverse, and can be presented quickly, clearly and smoothly on multiple screens at the same time; (2) The comfortable surrounding and space layout can enhance learning engagement and optimize the learning experience; (3) Learners and teachers can access and download rich digital resources through multiple channels at any time; (4) Learning context-aware is intelligent, which can capture, identify and record learners’ learning and psychological conditions, and promote personalized learning; (5) The interaction between learners and teachers, learners and learners, and human-machine would be facilitated; (6) Real-time feedback enables teachers to recognize learners’ learning achievements more effectively, so as to make more reasonable classroom adjustments, and can also provide timely feedback for learners based on the results of the provided assessment; (7) Learning communities will be connected, to form learning groups or teams, and to promote collaborative learning; and (8) Learning process will be recorded, which is a good way for learners to reflect on their learning process and find out the problems in learning.

## Methodology

The data in this study was collected through two methods: a comprehensive literature review and an expert survey. Specifically, as a first step, the findings about GBL problems were first collected from the literature based on a comprehensive literature review. Then, to further increase the validity of the constructed GBL model, it was reviewed and validated by experts using Delphi method. Each of the methods (literature review and Delphi) are discussed in the following sections.

### Literature Review

This review followed Kitchenham and Charters’ guideline for performing a systematic literature review (Keele, 2007) and was carried out through three phases: search strategy design, study selection, data extraction and data synthesis. Using literature review, this study identified some of the common teaching and learning problems in GBL and the affordance of smart classrooms for solving the problems.

#### Search Strategy

The search was conducted in databases that are well-known and well established in the field of education: Web of Science, EBSCO ERIC, IEEE Xplore and SpringerLink.

The search terms were constructed by Boolean logic as follows: “game-based learning” OR “gamification learning.”

In a pilot search, it appeared that the search engines of different databases use different syntax for search strings. Therefore, the search terms were adjusted to accommodate different databases.

#### Study Selection

The selection process consisted of two stages. The first stage was a preliminary screening, focusing on the following exclusion criteria.

• Studies which are published before 2010. This was because the term smart classrooms started to emerge in 2010.
• Studies without an abstract or in forms other than a paper (such as a poster, presentation, idea paper, etc.).
• Studies that did not elaborate on the research method used or the obtained findings.
• Studies that are not peer-reviewed.
• Studies which are not written in English.

The search term (“game-based learning” OR “gamification learning”) in the databases generated 1106 articles (Web of science:380; EBSCO ERIC:420; IEEE Xplore:252; Springer Link:54). The screening in previous stage excluded 562 articles and 544articles remained. After removing duplicate articles, 383 articles basically meet the requirements.

**FIGURE 1:**
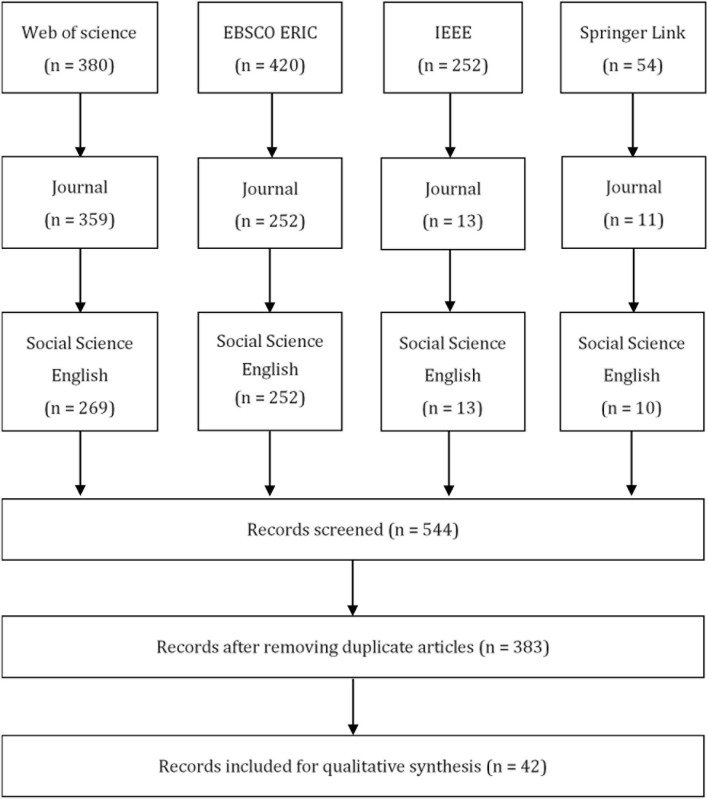
Review process.

Then, each study was downloaded in the second stage selection, where several selection criteria (see Table 1) where used to identify the relevance of each study to the research questions. The application of inclusion and exclusion criteria eliminated 383 articles, leaving 42 eligible studies (see Figure 1).

**TABLE 1:** Selection criteria in the second stage.

Inclusion criteria	Exclusion criteria
Research involves the background, conceptual interpretation or significance of GBL	Research that does not involve the background, conceptual interpretation or significance of GBL
Study points out the difficulties of implementing GBL in classrooms	Study that does not point out the difficulties of implementing GBL in classrooms

#### Data Extraction

An Excel form was designed to aid data extraction (Table 2). Each study was analyzed to derive these data, most of which were briefly presented in the results section. The analysis primarily focused on the problems of implementing GBL in classrooms.

**TABLE 2:** Coding sheet.

Database	Author(s)	Location	Title of publication	Year of publication	Type of article	Problems of implementing GBL in classrooms	Ref. (DOI/URL)

#### Data Analysis

This study adopted inductive content analysis (Elo and Kyngäs, 2008) to identify the problems of implementing GBL in classrooms in the selected studies. The steps were: selecting the unit of analysis, making sense of the data and the whole, open coding, coding sheets, grouping, categorization, abstraction, and conceptual mapping.

This study arranged two researchers of this paper for the coding. Two coders performed a pilot analysis on five papers together in order to reach agreement on the semantics of “problems of implementing GBL in classrooms.” Despite the inductive nature of this analysis, the coders used related literature as a reference (Lee et al., 2013; Tahir and Wang, 2020). Open coding allowed the possibility of collecting, analyzing and categorizing other problems.

### Expert Survey (Delphi Method)

A Delphi survey with GBL experts was conducted via email. Before the survey, experts were first contacted to check their interest in participating in this research. Additionally, the authors explicitly informed the experts that their participation would be anonymous. The experts were chosen based on their profiles, which should include: (1) GBL as their research interest; (2) good publication record in this area; (3) at least 5 years teaching experience.

As a result, 21 experts participated in this research (84% of active response), including scholars engaged in GBL research, and teachers who use GBL in their teaching. Despite that the experts were carefully chosen for this study to ensure the reliability of the findings, we further asked them to rate their familiarity with GBL, on a scale from 1 to 5 (where 1 is not familiar and 5 very familiar), as well as to write down their teaching experience in years. The experts had an average of 3.8 related to the familiarity with GBL, which reflect their high level of expertise and appropriateness for this study. The experts also had an average of 13 years as a teaching experience.

In the survey, the experts were requested to: (1) score 1–4 on the 25 elements extracted from the model (1 means not appropriate, 4 means very appropriate); (2) add GBL elements deemed necessary; (3) and give corresponding explanations for the choices they made. After the Delphi, we comprehensively analyzed the opinions of experts and modified the model.

## Results

### Problems for Implementing Game-Based Learning in Classroom (Research Question 1)

Based on the conducted literature review, the following problems for applying GBL in classroom were often found. Table 3 lists the difficulties of implementing GBL in traditional classroom mentioned in the reviewed papers. These items are classified from five aspects: infrastructure, resources, theoretical guidance, teacher’s capabilities and acceptance of GBL. The classification in Table 3 mainly relies on induction, but at the same time, it also refers to some related theoretical literature (Lee et al., 2013; Tahir and Wang, 2020).

**TABLE 3:** The difficulties of implementing GBL in classrooms.

Category	Description	Number of papers	Ref. (DOI/URL)
Infrastructure	The classroom hardware and software infrastructure is backward	4	10.1007/s10956-015-9571-7 10.3991/ijet.v14i16.10701 10.4018/ijgbl.2015010104 10.1016/j.chb.2011.11.007
	The constraints of inadequate and inappropriate technologies	5	10.1007/s11423-017-9552-z 10.1007/s10956-015-9571-7 10.1016/j.compedu.2019.04.016 10.1080/1369118X.2013.808365 10.1080/13603116.2014.885592
Resources	The quantity and quality of educational games need to be further improved	10	10.1007/s11423-017-9552-z 10.1016/j.chb.2020.106432 https://www.jstor.org/stable/jeductechsoci.17.1.42 10.1111/bjet.12346 10.1080/09639284.2016.1241951 10.3390/su12208487 10.1080/1369118X.2013.808365 10.1007/s40692-014-0008-8 10.1007/s40692-020-00174-5 10.1007/s10956-013-9436-x
	Lack of instructor-oriented authoring tools for educational games	3	10.1016/j.compedu.2018.09.011 10.1109/TLT.2011.1 10.1007/978-3-319-60291-2_14
Theoretical guidance	Lack of suitable frameworks on GBL within the curriculum	5	10.1111/bjet.12582 10.4018/ijgbl.2015010101 10.1080/09523987.2011.632277 https://www.researchgate.net/publication/343228250 10.4018/ijgbl.2015010104
	More appropriate instructional support needs to be designed to integrate games and teaching	5	10.3991/ijet.v8i6.2918 10.1007/s40299-019-00486-w 10.1007/s11528-015-0019-y 10.3390/educsci10090221 10.1109/TLT.2013.2294806
Teacher’s capabilities	Teachers’ instructional design ability needs to be improved	5	https://www.jstor.org/stable/26458512 10.1111/jcal.12438 10.1016/j.chb.2019.05.020 https://www.researchgate.net/publication/343228250 10.4018/ijgbl.2015010104
	Teachers’ technical literacy and organizational capabilities need to be improved	3	10.3991/ijet.v9i3.3294 10.1007/s10956-015-9571-7 10.4018/ijgbl.2015010104
	Teachers need to increase the knowledge of GBL	3	10.1.1.593.1566 10.1080/09585176.2015.1018915 10.4018/ijgbl.2015010104
Acceptance of GBL	Teachers’ acceptance of GBL	3	10.1016/j.compedu.2013.02.010 10.1016/j.compedu.2017.03.008 10.1007/s00530-009-0174-0
	Learners’ acceptance of GBL	2	10.1177/0735633119887187 10.1111/bjet.12314
	Parents’ acceptance of GBL	2	10.1016/j.compedu.2010.12.012 10.4018/ijgbl.2015010104

It should be noted that the total number of papers in Table 3 is more than the number of papers obtained by the final screening mentioned in the research method. This is because some papers have pointed out more than one type of problem, so they will be counted twice (or more) in Table 3. To summarize, the following problems were identified when using GBL in traditional classrooms.

(1) Digital educational games are more and more diversified, and the technologies used are more and more advanced. If teachers want to use these games to carry out GBL, they need to equip the corresponding technology and tools. However, many studies have pointed out that some of the present classroom hardware infrastructure could not support the needs of GBL, as some games with three-dimensional graphics interface have higher requirements on the central processor, memory and display card of the calculator (Nanayakkara and Whiddett, 2005; Webb et al., 2015). Traditional classrooms may be difficult to meet the needs of GBL activities. With the emergence, development and maturity of various intelligent technologies such as artificial intelligence, big data analysis, sensing technology, communication technology, cloud computing and the Internet of Things, GBL is increasingly used. The teaching practice of integrating new technologies requires a more complete learning space based on hardware facilities. It is important to establishing the infrastructure to enable gaming session (Marklund and Taylor, 2016). Therefore, one of the foundations of GBL is to have a good teaching environment, which requires appropriate technical environment to provide corresponding support.

(2) The lack of GBL resources is another major problem. The quantity and quality of educational game products need to be further improved (Sun et al., 2008). GBL needs to be based on GBL resources, such as high-quality digital games and related GBL products. However, when teachers adopt the GBL pedagogy, it is difficult for them to find the quality digital educational games. Some related enterprises and universities have begun to pay more attention to the production and development of GBL resources, and gradually strengthen the production, teaching and research integration of educational game resources development projects (Larsen, 2018; Lämsä et al., 2018; Gerodetti and Nixon, 2019; Romero et al., 2019). It will be a key research direction that can strengthen the construction of digital educational game resources, lower the threshold of GBL, and provide schools and teachers with richer products and more diversified choices. In addition to good games, the development tools for games are also what teachers need. But for now, there is still a lack of instructor-oriented authoring tools for educational games (Torrente et al., 2008; Paternò and Wulf, 2017; Brezovszky et al., 2019). It is therefore difficult for teachers to independently develop games suitable for teaching to implement GBL.

(3) There are still relatively few direct guiding theories that have a high degree of relevance for GBL. And there are few pedagogical models available for teachers who are interested in GBL (Denham, 2019). This is a major difficulty for teachers to implement GBL in classrooms. Without the guidance of proper theoretical framework, teachers may feel confused about how to apply games, what teaching activities to apply games in, how to arrange game time and learning scaffolds, how to integrate games into teaching and so on (Fang and Strobel, 2011). Not having a clear framework on GBL within the curriculum to guide teachers in the classroom, lack of subject knowledge and not knowing how to adopt new pedagogical approaches made it difficult for teachers to use games in teaching, and it also impacted on their view of teaching with games (Allsop and Jessel, 2015). Many studies have shown that it is very necessary for teachers to give them relevant theoretical guidance and instructional support (Denham et al., 2016; Belova and Zowada, 2020; Chen et al., 2020).

(4) Teachers’ information literacy and GBL design capabilities need to be improved (Becker, 2007). GBL should use some software and digital games, and therefore teachers need to enhance their information literacy so that they can be able to create digital learning environments. In GBL, there are often practical problems such as insufficient integration of games and learning content, game activities deviating from learning goals, low learner participation and so on. A survey conducted in 2013, where 488 teachers were asked questions to figure out what barriers hindered them from using games in the classroom, showed that 33% of the teachers found it was difficult to integrate games into the instruction (Fishman et al., 2014). Teachers should have good information literacy to successfully blend games with instruction, and they should also have good background about educational games to solve the technical problems that may arise in the process of teaching, and to provide timely and reasonable guidance for learners. In summary, teachers should strengthen the integration of GBL and classroom teaching, which means they need to do better in optimizing instructional design, developing diversified teaching evaluation methods, supporting learners’ individualized learning and creating teaching situations (Becker, 2007; Denham et al., 2016).

(5) The acceptance of GBL is another realistic issue in the implementation of GBL. The adoption and the effectiveness of GBL depend largely on the acceptance by classroom teachers, as they can be considered the true change agents of schools. Research surveys have shown that teachers’ perceptions of video games are complex. On the one hand, teachers are not really convinced that video games are very useful for enhancing their job performance. On the other hand, they believe that video games provide opportunities for learning (Bourgonjon et al., 2013; Huizenga et al., 2017). From the perspective of students, they may have a relaxed and entertaining attitude toward playing games, while ignoring the purpose of learning (Israel-Fishelson and Hershkovitz, 2020). Their level of interest in the game and the duration of the operation are also different, which will affect the participation of students (Ke et al., 2016). From the perspective of parents, they are more concerned about whether children can form a better balance between play and study life (Bourgonjon et al., 2011; Vate-U-Lan, 2015). However, what we can expect is that with the development of GBL, people’s acceptance of GBL will gradually increase, and more relevant groups will have a positive view of it.

### The Technology Enhanced Game-Based Learning Model (Research Question 2)

In the information age, emerging technologies could be used to help teachers implement GBL better. With the advance of educational technology, the research and practice of smart classroom became popular (Yang et al., 2018), to facilitate content presentation, class management, learning resources accessing, and instructional interaction by utilizing appropriate devices and software (Huang et al., 2012). Some studies point out the characteristics of smart classrooms include both virtual and physical environments (Rogers, 2002), provide access to data to facilitate learners’ investigations (Clark et al., 2007), and produce relevant feedback for learners (Balacheff et al., 2009). By summarizing and sorting out relevant literature, this study extracts eight elements of smart classrooms:

In order to solve some of the problems (such as: infrastructure, resources, and theoretical guidance) in GBL by making good use of technology, and combined with relevant literature, this study constructs the technology enhanced GBL model. The design of GBL process in this model mainly refers to the input-process output model (Garris et al., 2002). In addition, some ideas from The Play Curricular Activity Reflection and Discussion (PCaRD) GBL pedagogical model (Denham, 2019), and the ARCS model (Keller, 1987) are also used for reference. In order to verify the validity of the model, this study adopted the Delphi method. There are 25 key elements that can be extracted from the model. All the elements of the model were identified based on a comprehensive literature review. The results of the degree of acceptance of each element of the model based on the experts’ rating are shown in Table 4.

**TABLE 4:** Results of the degree of acceptance about elements of the model (the technology enhanced GBL model) based on the experts’ rating.

	Element	Mean	*SD*	Coefficient of variation
1	Connecting learner’s community	3.48	0.81	0.23
2	Intelligence test and data acquisition	3.57	0.68	0.19
3	Real-time feedback	3.71	0.64	0.17
4	Personalizing learning pace	3.57	0.68	0.19
5	Convenient learning facilities	3.52	0.60	0.17
6	High speed Internet access	3.57	0.51	0.14
7	Comfortable surroundings	3.00	1.00	0.33
8	Flexible space layout	3.19	0.75	0.23
9	Multimodal learning analysis	3.71	0.56	0.15
10	Pre-analysis	3.33	0.80	0.24
11	Game selection	3.62	0.74	0.20
12	Context design	3.76	0.44	0.12
13	Activity design	3.95	0.22	0.06
14	Learning contents	3.43	0.75	0.22
15	Features of the Game	3.57	0.81	0.23
16	Gamification of learning contexts	3.76	0.44	0.12
17	Thinking and inspiration	3.43	0.81	0.24
18	Gamification exploration	3.48	0.87	0.24
19	Collaboration and communication	3.62	0.50	0.14
20	Presentation and sharing	3.38	0.67	0.20
21	Learning outcomes	3.38	0.74	0.22
22	Assigning homework	2.95	0.67	0.23
23	Personalized guidance	3.71	0.46	0.12
24	Reflection and improvement	3.43	0.75	0.22
25	Next round planning	3.19	0.81	0.26

**FIGURE 2:**
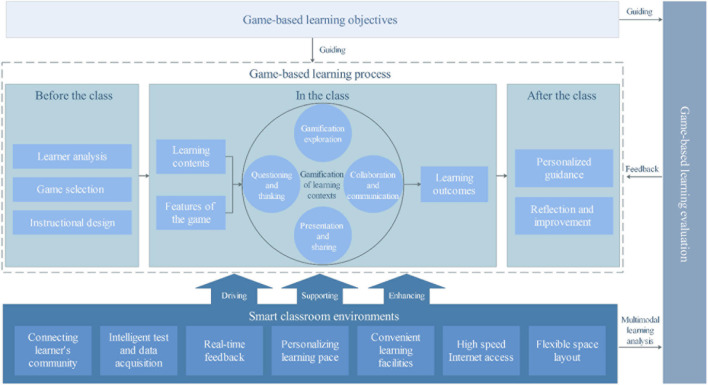
The technology enhanced GBL model.

There are a total of 21 samples in round 1, 25 items in each questionnaire. The questionnaire’s Cronbach’s alpha is 0.916, and the Cronbach’s alpha of each item in the questionnaire is greater than 0.9. This means that the questionnaire’s reliability is high, and the collected data are reliable. The Mean score represents the expert’s recognition of the elements. In this study, items below 3 are deleted. A score of 3 or below indicates that experts did not have high acceptance level toward the given element, so “22 Assigning homework” was deleted. Coefficient of Variation indicates the degree of coordination of expert degree of acceptance of the elements, the smaller the coefficient, the higher the degree of coordination of experts. It is generally believed that CV < 0.25 is a good indicator. And this study will delete items with CV ≥ 0.25, so “7 Comfortable Environment” and “25 Next Round Planning” were deleted. In addition, combined with the qualitative evaluation of experts, some elements are modified. And the final technology enhanced GBL model in this study is shown in Figure 2.

The technology enhanced GBL model is mainly composed of four parts: smart classrooms, GBL objectives, GBL process, and GBL evaluation. The learning objectives of a class need to be achieved through the dynamic interaction of teaching/learning and evaluation. Teachers should prepare for the GBL process by considering the real-time feedback to design the context, choose games, and guide learning activities. Using different technologies in the learning environment can accessing digital game resources, timely test and feedback, displaying learning analytic infographic, etc. for driving teacher’s instructional design, supporting learning activities, and enhancing communications between teachers and students.

## Discussion, Conclusion, and Future Research

This study focused on the problems faced by GBL in the implementation process, and attempts to find ways to deal with some of these problems from the perspective of using technology. The study found that there were five common problems in the implementation of GBL in the classroom: (1) the backward classroom infrastructure with inadequate and inappropriate technologies, (2) the lack of quality educational game resources and instructor-oriented authoring tools, (3) the weak theoretical guidance of frameworks, curriculum and instructional support, (4) the incompetence of teacher’s information literacy for GBL, (5) the stakeholder’s hesitation in adopting GBL.

Based on the experts’ inputs using the Delphi method, the eight elements of connecting learner’s community, intelligence test and data acquisition, real-time feedback, personalizing learning pace, convenient learning facilities, high speed internet access, comfortable surroundings and flexible space layout of smart classrooms were identified (Kariippanon et al., 2020; Midcalf and Boatwright, 2020; Wang et al., 2021, p. 19). Combined with the elements and the general process of GBL, the technology enhanced GBL model was constructed. This model consisted of four parts: GBL objectives, GBL process, GBL evaluation and smart classrooms. The model explains the process and main activities of GBL from the three stages of before the class (Becker, 2007; Huang et al., 2019), in the class (Garris et al., 2002; Uzelac et al., 2015; Denham et al., 2016; Paudel et al., 2020; Kim et al., 2021) and after the class (Bayirtepe and Tuzun, 2007; Suo et al., 2008; Yang and Huang, 2015; Aguilar et al., 2020).

The design and formulation of the model can also respond to the lack of theoretical guidance to a certain extent.

(1) For the problem of infrastructure, this model provides a method for constructing suitable environments for GBL. The environments should have high-speed Internet access, which makes the game process and communication smooth. Convenient learning facilities include various types of learning terminals, display terminals, and real recording terminals, which can effectively support the presentation and sharing of learning content and learning results, and support the communication and interaction between teachers and learners. The flexible spatial layout is mainly to provide learners with a more open venue for activities, rather than confining the space for teaching activities to closed conventional rooms. It is convenient for teachers to arrange the seats of students according to different game forms and teaching activities. Desks and chairs with humanized designs are provided so that learners can change their positions according to their needs, and form learning groups to facilitate teamwork and group learning activities.

(2) For the problem of theoretical guidance, this model provides guidance for teachers’ to implement GBL activities. Using this model, teachers who do not know how to implement GBL can first have a clear cognition of GBL, and can understand the general process of GBL. In addition, teachers with GBL experience may be able to make some new discoveries and try to optimize learning analysis, learning activities and learning evaluation by using various technologies in the learning environment. This can help them to attract learners’ interest and promote learners’ effective learning. Therefore, the proposed model gives teachers some guidance in theory.

However, the model could not handle the other three problems of resources, teacher’s capability and acceptance to GBL, which could be targeted in future studies.

The model could be used by researchers, teachers, and school administrators, or other stakeholders. For researchers, it can serve as a reference for further research on implementing GBL in smart classrooms, because in the increasingly intelligent environment, GBL will develop to a new stage, which requires researchers to carry out research to keep pace with the times. For teachers, it provides a guidance on implementing GBL in smart classrooms, because the model proposed in this paper is mainly designed according to the teaching process, teachers can refer to it in different teaching links.

(1) Before the class, teachers can choose the appropriate games and design teaching activities. Teachers can also design realistic and interactive game-based learning situations.

(2) In the class, teachers can create immersive GBL experience that can evoke thinking, promote learning by exploring through different game activities, as well as develop collaborative capability and improve interpersonal communication skills. Encouraging presentation and sharing, learners share their learning results with others and display their works through various content presentation methods in the smart classroom, such as multi-screen display and file transfer between terminals.

(3) After the class, teachers can monitor the online learning process to better enhance learning and improve the quality of teaching. Enhancing personalized guidance, where teachers can find students who have difficulty in learning by viewing and analyzing the student data collected by in the learning process. Teachers provide targeted guidance to learners to solve students’ learning difficulties. Boosting reflection and improvement, where teachers reflect on the effects of teaching, redesign and improve the deficiencies. Teachers can get enlightenment from the reflection, which can become the experience and basis for teachers to improve their teaching ability.

Although the previous research basis on smart classrooms and the systematic literature review on GBL provided solid foundation for the reliability of the proposed model, this model was still in the stage of theoretical conception and had not been applied in practice. However, the idea of this model was presented at an international conference on GBL, where lots of teachers expressed that they were inspired and were willing to carry out relevant practice. Besides, the study also verified the validity of the model through the Delphi method.

This study mainly constructs a GBL model supported by smart classrooms from a theoretical perspective, however it must take further exploration in the educational field to enhance the validity. It is promising that in the near future, the integration of the GBL and smart classrooms will be explored in-depth from both theoretical and practical perspectives.

## Data Availability Statement

The original contributions presented in the study are included in the article/supplementary material, further inquiries can be directed to the corresponding author/s.

## Author Contributions

LP and JY: conceptualization. JY, JL and FJ: methodology. HY and JY: supervision. JY, HY, JL, and GS: resources. LP, HY, and JY: investigation, data curation, writing—original draft preparation. AT, JY, HY, JL, FJ, and GS: writing—review and editing. All authors have read and agreed to the published version of the manuscript.

## Conflict of Interest

The authors declare that the research was conducted in the absence of any commercial or financial relationships that could be construed as a potential conflict of interest.

## Publisher’s Note

All claims expressed in this article are solely those of the authors and do not necessarily represent those of their affiliated organizations, or those of the publisher, the editors and the reviewers. Any product that may be evaluated in this article, or claim that may be made by its manufacturer, is not guaranteed or endorsed by the publisher.
